# Bioinformatics analysis identifies dysregulation of miR-548F-3p and its hub gene in triple-negative breast cancer

**DOI:** 10.22038/ijbms.2025.79808.17287

**Published:** 2025

**Authors:** Samira Behroozi, Mahdieh Salimi, Najaf Allahyari Fard

**Affiliations:** 1 National Institute of Genetic Engineering and Biotechnology (NIGEB), Institute of Medical Biotechnology, Department of Medical Genetics,; 2 Tehran, Iran; 3 National Institute of Genetic Engineering and Biotechnology (NIGEB), Tehran, Iran

**Keywords:** Computational biology, Gene expression profiling, MicroRNAs, Survival analysis, Triple-negative breast - neoplasms

## Abstract

**Objective(s):**

Triple-negative breast cancer (TNBC), which affects 15–20% of cases, lacks targeted therapies and poses challenges in treatment. MicroRNAs (miRNAs) are potential biomarkers and therapeutic targets in breast cancer. To unravel its unique regulatory role, this study focused on miRNA microarray analysis, particularly miR-548F-3p, in TNBC samples.

**Materials and Methods:**

Using the GSE76275 dataset, gene expression profiles were analyzed using the Affymetrix Human Genome U133 Plus 2.0 Array. Differentially expressed genes (DEGs) were identified using robust preprocessing. Weighted gene co-expression network analysis (WGCNA) explored gene modules and identified hub genes co-expressed with miR-548F-3p. Functional enrichment and protein-protein interaction (PPI) network analyses were conducted. Survival analysis was used to assess the prognostic impact of the identified genes.

**Results:**

The study found 224 up-regulated DEGs, with miR-548F-3p exhibiting significant down-regulation. MultimiR identified 400 genes that were targeted by miR-548F-3p. WGCNA revealed a blue co-expression module, with 356 genes targeted by miR-548F-3p. A Venn diagram identified common genes, including VANGL2, BRCC3, ANP32E, and ANLN. Functional enrichment highlighted crucial pathways in TNBC pathogenesis, including mitotic spindle organization, spindle assembly checkpoint signaling, cell cycle, and amino acid (serine) metabolism. PPI network analysis identified hub genes, including FOXM1, KIF23, and CDC20. VANGL2, BRCC3, ANP32E, and ANLN were significantly associated with patient outcomes in survival analysis.

**Conclusion:**

This analysis highlighted TNBC’s molecular landscape, emphasizing miR-548F-3p’s regulatory role. The identified genes, VANGL2, BRCC3, ANP32E, and ANLN, offer insights into TNBC pathogenesis and potential therapeutic targets, laying the foundation for understanding their clinical implications in the intricate landscape of TNBC.

## Introduction

Triple-negative breast cancer (TNBC) is a very aggressive tumor that primarily affects young women. TNBC is often detected at a later stage and lacks specific treatment options (1). TNBC, which accounts for 15–20% of breast cancer cases, is a unique subtype characterized by the absence of estrogen receptors, progesterone receptors, and HER2 receptors on the cell surface (2). Analysis of gene expression patterns revealed that TNBC is categorized as a basal-like breast cancer subtype (3). Compared with other subtypes of breast cancer, TNBC often develops in young women and is associated with higher levels of aggressiveness and death (4). Approximately 46% of individuals diagnosed with TNBC experience distant metastases occurring in either the brain or other regions of the body (5). Consequently, the average survival duration was found to be approximately 13.6 months (6). Multiple investigations have shown that as many as 25% of patients diagnosed with TNBC have the potential to achieve recovery. The utilization of anti-metabolites, paclitaxel, and anthracyclines in adjuvant and neoadjuvant chemotherapy for individuals diagnosed with TNBC has been approved by the Food and Drug Administration (FDA) (7). Regular chemotherapy has shown a degree of efficacy in individuals diagnosed with TNBC. Nevertheless, the deleterious effects of chemotherapy pose a significant risk to patients; regrettably, there are instances when patients fail to derive any therapeutic advantages. Hence, identifying optimal targets for the precise treatment of TNBC is a complex and crucial therapeutic issue that requires resolution (8). MicroRNAs (miRNAs) are short RNA molecules, approximately 20–25 nucleotides long, that naturally occur within an organism and do not code for proteins. They regulate gene expression after transcription by removing targeted mRNA or inhibiting its translation. This regulation occurs through miRNA binding to their target mRNAs’ 3′ untranslated region (3′UTR) (9). miRNAs play crucial roles in multiple biological processes, such as cell proliferation, death, and development (10). It is essential to acknowledge the importance of genetic variables, including genes and miRNAs, in understanding and tackling the resistance of TNBC to traditional chemotherapy. This recognition is essential for developing targeted and effective treatment approaches to improve patient outcomes.

The swift progress of microarray and next-generation sequencing technology allows researchers to discern changes in gene expression data across various cancer types (11). Weighted gene co-expression network analysis (WGCNA), commonly known as weighted correlation network analysis, is a systematic biological approach used to characterize the correlation of gene expression across multiple datasets. This method has been widely applied to unveil highly pertinent gene clusters, known as modules, and identify potential hub genes by evaluating the connections among gene modules and the correlation between gene modules and clinical characteristics (12).

This study used publicly accessible TNBC datasets. After identifying genes with altered expression and miRNAs showing significant changes, further investigation into their expression was conducted. Using WGCNA, genes co-expressed with the specific miRNA of interest were methodically identified. Gene enrichment was explored, and protein-protein interaction (PPI) networks were created. We aimed to understand the complex molecular interactions within TNBC to gain valuable insights into the regulatory networks of genes and miRNAs.

## Materials and Methods

### Gene microarray data

In this study, gene microarray data were obtained from the GSE76275 dataset of the Gene Expression Omnibus (GEO) database, chosen for its comprehensive representation and relevance to the research focus. The dataset was selected because of its extensive coverage of 256 samples encompassing diverse breast cancer subtypes, including 198 cases of TNBC. The dataset includes detailed patient information, such as tissue type, sex, race, body mass index (BMI), and menopausal status, along with histology group classifications and specific breast cancer types. It also provides essential data on the estrogen receptor (ER), progesterone receptor (PR), and HER2 status, which are crucial for treatment implications. Additionally, it confirms triple-negative status and includes measurements of tumor size, positive lymph nodes, and metastasis presence at diagnosis.

This broad representation ensures that the analysis captures the spectrum of gene expression patterns across various breast cancer phenotypes, thereby enhancing the robustness and applicability of the findings to research objectives. Our study focused on comparing gene expression profiles among the categories above. The Affymetrix Human Genome U133 Plus 2.0 Array (GPL570) was utilized to generate the microarray data, which enabled an exhaustive examination of the gene expression patterns linked to TNBC.

### Data preprocessing and differentially expressed genes (DEG) Identification

**Figure 1:**
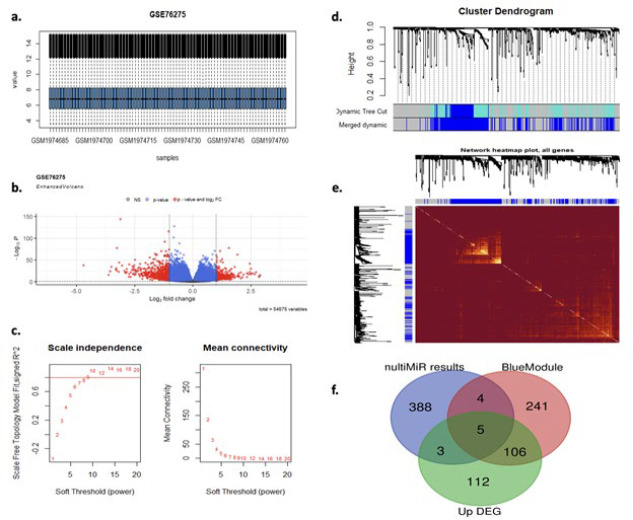
Integrated analysis of gene expression and co-expression networks in TNBC

Gene microarray data were obtained from GEO and preprocessed using R software version 4.3.2. A robust multichip average (RMA) application was utilized for background correction, normalization, and probe summarization. The limma (Linear Models for Microarray Analysis) application was used to identify DEGs between TNBC and other breast cancer subtypes after normalization. Limma is a powerful tool known for its robust statistical methods and flexibility in analyzing microarray data and accurately identifying gene expression changes. The preliminary examination utilized a rigorous criterion, which included a log-fold change (logFC) of no less than 1 or greater than -1 and an adjusted *P*-value (adj.P.Value) below 0.01. Following that, to visually evaluate the efficacy of the preprocessing procedures and detect possible outliers, box plot diagrams were constructed from the normalized data (Figure 1a). Volcano graphs were generated to depict the distribution of DEGs, emphasizing the correlation between logFC and statistical significance (Figure 1b). The miRNA exhibiting the most pronounced reduction was chosen as the target and targeted with the multiMiR package expands the analytical scope by extracting targets from mircode, mirTarbase, and Tarbase, enabling comprehensive investigation of potential regulatory networks associated with the identified DEGs, thereby enriching the understanding of molecular mechanisms underlying breast cancer subtypes.

### WGCNA analysis

After preprocessing the data, the expression matrix was processed using the WGCNA package with identical threshold criteria, as previously stated. WGCNA is a powerful systems biology approach that identifies modules of highly correlated genes across samples, facilitating the exploration of gene networks and their relationships with clinical traits. By leveraging network theory principles, WGCNA enables the identification of co-expressed gene modules, providing a holistic view of the underlying biological processes that drive breast cancer subtypes.

A systematic process was employed to eliminate outlier samples, and the absent values were filtered. The co-expression network structure and intended miRNA co-expression module were determined by constructing a similarity matrix among all genes using Pearson’s correlation coefficient. A lenient threshold of nine was established to satisfy the scale-free co-expression network relationship. Following this, the adjacency matrix was utilized to deduce the topological overlap matrix and to facilitate the hierarchical clustering of genes using dynamic cut-tree algorithms, and the corresponding dissimilarities were computed. Furthermore, the power threshold implemented during the WGCNA method, which is critical for establishing module relationships, is represented graphically on the ‘Soft power’ graph (Figure 1c). The process of integrating distinct modules based on high similarity is depicted in the ‘Merge’ graph (Figure 1d). The ‘Tomplot’ offers valuable insights into the topological characteristics of the co-expression network (Figure 1e).

### Gene functional annotation analysis

An exhaustive gene functional annotation analysis was performed to elucidate the biological importance of genes that were identified through GSE analysis, specifically those exhibiting heightened expression. The convergence of genes obtained from the multiMiR package, those predicted from the GSE analysis (up-regulated DEGs), and the desired miRNA co-expression module from WGCNA was the primary focus of our investigation. Convergence was symbolically represented through the utilization of a Venn diagram, which highlighted common elements among these unique gene sets. Following this, genes that appeared in at least two sets in the Venn diagram were selected for additional examination. Subsequently, a comprehensive functional annotation analysis was conducted using the EnrichR database. The scope of this investigation included a variety of Gene Ontology (GO) classifications, including Molecular Function (MF), Biological Process (BP), Cellular Component (CC), and pathways obtained from the WikiPathways database.

### PPI network analysis and hub gene identification

A comprehensive PPI analysis was performed to clarify the potential connections. This involved exploring shared genes among the up-regulated DEGs, the identified miRNA co-expressed module through WGCNA, and the predicted genes from the multiMiR package. Utilizing the Cytoscape software (V. 3.10.1), a systematic mapping of common genes onto the PPI network was conducted. Subsequently, Cytohubba, a Cytoscape plugin known for its ability to identify key hub genes within biological networks, was employed to systematically select the top 10 hub genes based on their connectivity within the PPI network, thereby providing insights into potential central players regulating biological processes associated with breast cancer subtypes. 

### Survival analysis

To examine the prognostic significance of the identified genes (ten hub genes and five shared genes in three gene lists), a survival analysis was conducted using the ULACAN online database (13), which incorporated clinical data from GSE76275 breast cancer patients. ULACAN offers a user-friendly interface and comprehensive analysis tools to explore the association between gene expression profiles and patient outcomes, thereby providing valuable insights into the prognostic relevance of the identified genes in the context of breast cancer. Relapse-free survival (RFS) and overall survival (OS) were assessed to determine the effects of gene expression on patient outcomes. Using the ULACAN platform, Kaplan-Meier survival curves were constructed in real-time, depicting the likelihood of survival for unique gene expression groups. The statistical significance of survival differences between these groups was determined using the log-rank test. Genes that exhibit a substantial correlation with survival outcomes may be recognized as prospective prognostic indicators, yielding valuable insights into their potential clinical applicability in breast cancer prognosis.

## Results

### Gene microarray data, data preprocessing, and WGCNA analysis

We identified 224 differentially expressed genes (DEGs) that were up-regulated in our exhaustive analysis of the gene microarray data obtained from the GSE76275 dataset. We used stringent criteria, including a log-fold change (logFC) of at least ±1 and an adjusted *P*-value (adj.P.Value) of less than 0.01. It is worth noting that miR-548F-3p exhibited the most significant reduction in expression among the miRNAs. Additional investigation using the multiMiR package revealed the identity of 400 predicted target genes for miR-548F-3p. Entertaining patterns were revealed in the co-expression network via WGCNA analysis. It is worth mentioning that the blue module, which contained 356 genes that exhibited co-expression with miR-548F-3p, contained the target miR-548F-3p. This discovery offers insights into possible regulatory networks linked to this miRNA. An integrative analysis was conducted on the up-regulated DEGs, genes from the desired miRNA co-expressed module obtained from WGCNA, and predicted genes from the multiMiR package, employing the Venn diagram methodology illustrated in Figure 1f. This analysis identified 118 genes shared by at least two gene collections. It is noteworthy that five genes, specifically Solute Carrier Family 16 Member 1 (SLC16A1), BRCA1/BRCA2-containing complex 3 (BRCC3), SRY-Box Transcription Factor 4 (SOX4), Acidic (Leucine-Rich) Nuclear Phosphoprotein 32 Family Member E (ANP32E), and Van Gogh-Like Protein 2 (VANGL2), were discovered to be shared across all three gene collections. 

### Functional enrichment analysis of common genes

**Figure 2:**
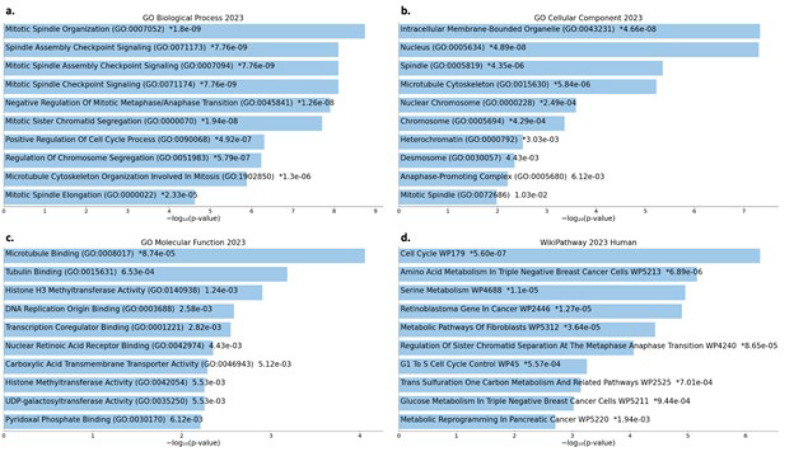
Functional and Pathway Enrichment Analysis of Shared Genes with a Bar Graph Representation of Significance (*P*<0.05) Across Biological Processes, Cellular Components, Molecular Functions, and Wikipath

An analysis of functional enrichment was conducted on the 118 genes present in a minimum of two gene lists. The results revealed a spectrum of biological processes implicated in the pathogenesis of breast cancer (Figure 2a), including negative regulation of metaphase/anaphase transition, spindle assembly checkpoint signaling, and mitotic spindle organization. Associations with intracellular membrane-bounded organelles, nuclei, spindles, microtubule cytoskeletons, and nuclear chromosomes were identified through cellular component analysis (Figure 2b). The roles of microtubule and tubulin binding, histone H3 methyltransferase activity, DNA replication origin binding, and transcription co-regulator binding were identified through molecular function annotations (Figure 2c). Significant involvement in critical pathways, including the cell cycle, amino acid metabolism in retinoblastoma cells, serine metabolism, the retinoblastoma gene in cancer, and metabolic pathways of fibroblasts, was identified through pathway enrichment (Figure 2d). 

### PPI network analysis and hub gene identification

**Figure 3:**
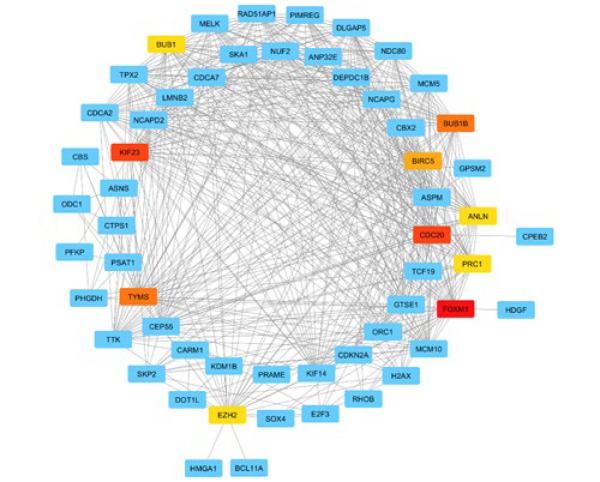
PPI network analysis. The PPI network resulting from the analysis consisted of 57 nodes interconnected by 542 edges, representing potential protein-protein interactions among the identified genes

**Figure 4:**
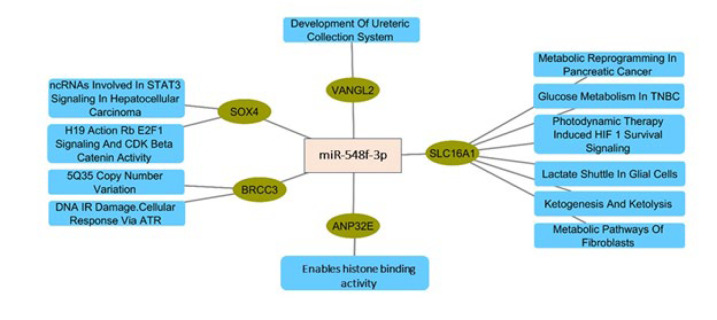
MiRNA-gene-pathway network for miR-548F-3p and common genes

An exhaustive PPI network analysis was performed to elucidate potential interactions between the 118 common genes identified from the up-regulated DEGs, the Desired miRNA co-expressed module from WGCNA, and the predicted genes from the multiMiR package. The Cytoscape software was utilized to map the shared genes methodically onto the PPI network. Subsequently, the degree method was employed by Cytohubba to rigorously ascertain the ten most significant hub genes within the network. As illustrated in Figure 3, the generated PPI network consisted of 57 interconnected nodes along 542 edges. This intricate configuration effectively depicts the potential protein-protein interactions that may occur among the identified genes. The main hub genes, namely Forkhead Box M1 (FOXM1), Kinesin Family Member 23 (KIF23), Cell Division Cycle 20 (CDC20), Thymidylate Synthase (TYMS), Budding Uninhibited by Benomyl 1 Homolog Beta (BUB1B), Baculoviral IAP Repeat Containing 5 (BIRC5), Budding Uninhibited by Benomyl 1 (BUB1), Enhancer of Zeste 2 (EZH2), Anillin Actin Binding Protein (ANLN), and Protein Regulator of Cytokinesis 1 (PRC1), were identified, indicating their critical roles and prominence in the network. Table 1 presents the expression details of the top ten hub genes. Furthermore, a separate network analysis was performed on miR-548F-3p and the five genes present in all three gene lists (Figure 4). 

### Survival analysis results

**Figure 5:**
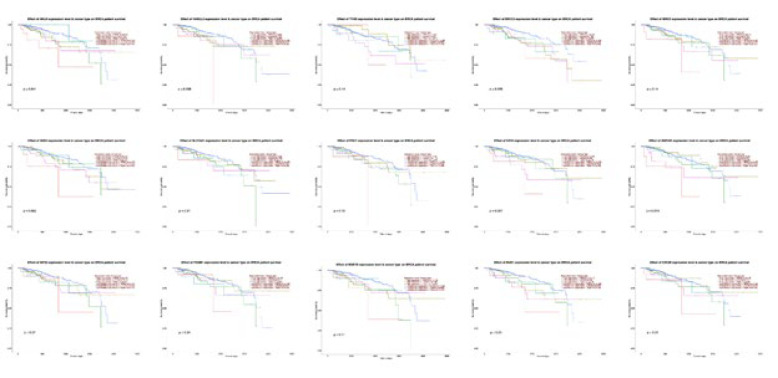
Survival analysis was conducted to investigate the prognostic implications of 15 genes, including the five shared genes identified in all three gene lists and the ten hub genes

**Figure 6:**
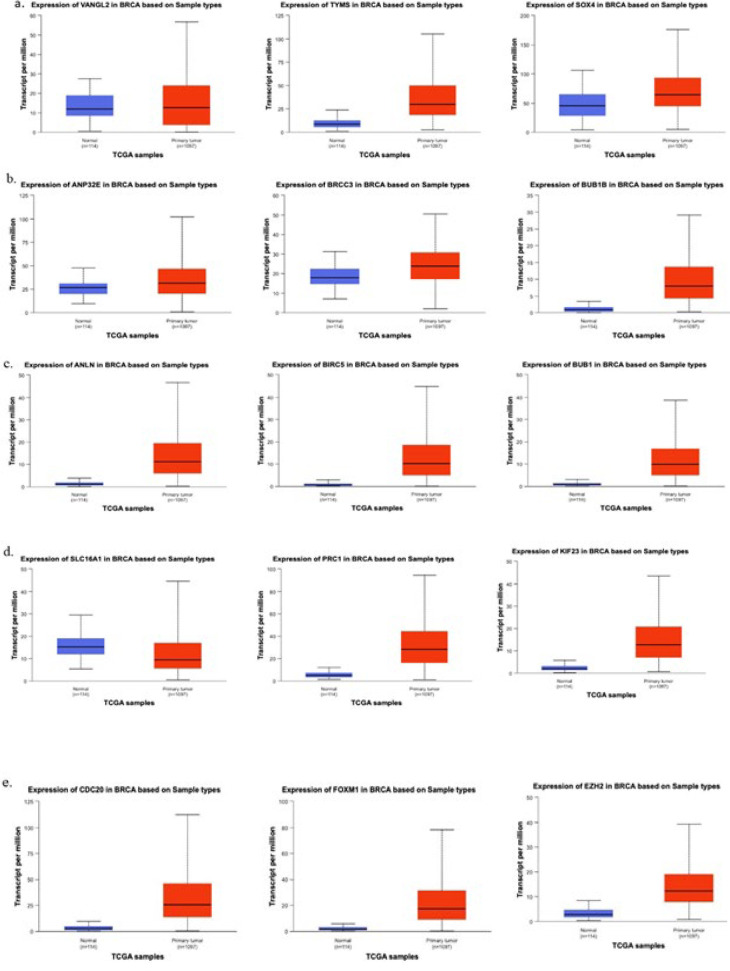
Expression of the desired gene in normal and primary tumor samples using TCGA database in ULCAN

The ULACAN survival analysis of breast cancer patients using the GSE76275 dataset revealed that 14 of the 15 genes analyzed exhibited up-regulated expression, while SLC16A1 was down-regulated. It is worth mentioning that areas shared by three gene lists (ANP32E), VANGL2, BRCC3, and ANLN (a hub gene), exhibited substantial correlations with patient outcomes, specifically OS and RFS (Figure 5). Based on TCGA datasets, Figure 6 depicts the expression levels of desired genes in normal and primary tumor samples. The results of this study highlight the possible prognostic significance of these genes in TNBC.

## Discussion

Breast cancer is recognized as a multifaceted and diverse illness characterized by different molecular subcategories and varying responses to treatment. Despite significant advancements in breast cancer treatment, including endocrine therapy and HER2-targeted therapy, there is currently a lack of molecularly targeted treatments for patients with TNBC. Recent research has shown that miRNAs might be used as biomarkers and targeted for therapy in breast cancer (14), with the potential to influence disease progression, stage, and genetic predisposition (15). The progression of TNBC is often marked by increasing aggressiveness and metastatic potential; for example, studies have shown that elevated levels of miR-21 correlate with advanced disease stages and poorer outcomes (16). The stage at which TNBC is diagnosed significantly influences treatment choices and patient prognosis. For example, a specific miRNA profile consisting of six distinct miRNAs (miR-21, miR-221, miR-210, miR-195, miR-145, and let-7a) has been linked to advanced stages, elevated tumor grades, and negative hormone receptor status in Indian women with TNBC (17). Furthermore, genetic predisposition plays a vital role in TNBC, as specific miRNA variants—such as the miR-423-5p AC genotype found in European populations—are linked to a higher risk of breast cancer compared to the CC and AA genotypes seen in Asian and African women, respectively (18).

This study performed miRNA microarray analysis on TNBC samples compared to other breast cancer forms, enhancing our understanding of the complex molecular landscape of the disease. We focused on miR-548F-3p, a novel miRNA that appears particularly relevant to TNBC. Located on the X chromosome at Xp21.1 (19), miR-548F-3p offers unique insights into the regulatory processes driving TNBC. The expression profile illustrated in the human GeneAtlas U133A (20) reveals miR-548F-3p activity across various organs, with the highest expression levels found in the retina, followed by the cerebellum and cerebellar peduncles. Additionally, this study innovatively integrates advanced bioinformatics techniques, including WGCNA and PPI networks, to elucidate the regulatory networks involving miR-548F-3p and its target genes in TNBC, thereby establishing a comprehensive framework for potential therapeutic interventions.

Understanding the pathways driving TNBC aggressiveness and the role of targeted molecules are essential for effective treatment strategies. Key pathways include cell cycle regulation with overexpressed cyclin-dependent kinases (CDKs), leading to unchecked cell division (21); the PI3K/AKT/mTOR pathway, which is hyperactivated, promoting tumor growth and therapy resistance (22); and DNA repair defects involving BRCA1/2 (23) and PARP (24), making tumors more susceptible to specific treatments. Additionally, pathways such as androgen receptor signaling and RAS/MAPK contribute to tumor progression and present potential therapeutic targets (25). This comprehensive molecular understanding is critical for developing innovative treatment strategies, particularly combination therapies that can improve TNBC patient outcomes. Our functional enrichment analysis further elucidated the molecular foundations driving breast cancer pathogenesis by identifying the major biological processes involved in its growth and progression. The focus on mitotic spindle organization emphasizes the importance of precise cell division, which is required for proper chromosomal segregation during proliferation and, when dysregulated, is a marker of cancer-associated genomic instability and aneuploidy (26). Equally important is the focus on spindle assembly checkpoint (SAC) signaling, which emphasizes its precise regulatory mechanisms in guaranteeing mitotic spindle formation integrity. The involvement of SAC in delaying chromosome segregation until correct attachments are formed is critical, particularly in cancer cells where chromosomal instability (CIN) is prevalent and often caused by undiagnosed attachment defects (27). Defects in spindle formation, particularly those involving the microtubule-binding protein TPX2, lead to attachment errors and CIN, which correlates with the aggressiveness of many human tumors (28). Furthermore, identifying negative regulation of the metaphase/anaphase transition highlights the tight control exercised over cell development through the cell cycle phases, emphasizing its crucial function in maintaining genomic integrity. In the cancer context, techniques targeting the metaphase-anaphase transition show potential for developing cell cycle-selective therapeutics (29). Furthermore, data from a thorough study of 1135 breast cancer patients with up to a 22-year follow-up demonstrated the predictive value of immunoexpression for the combination of Securin and Separase, regulatory proteins involved in the metaphase/anaphase transition. This combination has emerged as an effective predictive tool for identifying individuals at risk of breast cancer-related death (30).

Furthermore, our comprehensive pathway enrichment analysis revealed a diverse landscape of the major molecular pathways intimately implicated in breast cancer, offering a better understanding of the underlying cellular processes. The strong participation in the cell cycle route emphasizes the importance of cell division control, validating the idea that dysregulation in this complex process, such as cell cycle-related enhanced biological processes, is a hallmark of cancer, as identified in other studies (31-33) utilizing identical methods. Aberrant cell cycle progression often leads to uncontrolled proliferation, a distinguishing feature of malignant cells (34). The discovery of amino acid metabolism in TNBC cells as a considerably enhanced pathway provides information on metabolic changes specific to this aggressive subtype. Amino acid metabolism is critical for cancer cell proliferation and survival, and dysregulation is becoming recognized as a defining trait in many cancer forms, including TNBC (35). In this context, Jeon et al. discovered that restricting methionine consumption inhibits tumor development and metastasis in TNBC (36). The predominance of serine metabolism revealed the possible metabolic weaknesses of the TNBC cells. Serine metabolism is recognized to be required for nucleotide synthesis and redox equilibrium, which are critical elements enabling cancer cells’ fast proliferation and survival (37). Notably, TNBC cells treated with doxorubicin underwent metabolic remodeling, resulting in enhanced serine synthesis mediated by phosphoglycerate dehydrogenase (PHGDH). Serine is then converted to glutathione, which inhibits the generation of reactive oxygen species generated by doxorubicin. As a result, inhibition of PHGDH may increase the sensitivity of cells to doxorubicin (37). This dynamic relationship between amino acid metabolism, especially serine metabolism, and chemotherapy response demonstrates TNBC’s complicated metabolic landscape and suggests possible therapeutic interventions.

In our investigation of TNBC, we examined the critical genes related to patient outcomes, including VANGL2, BRCC3, ANP32E, and ANLN. The VANGL2 gene produces a membrane protein that regulates planar cell polarity, notably in the cochlea’s stereociliary bundles (38). VANGL2 mutations cause severe developmental abnormalities, emphasizing their critical role in embryonic tissue organization (39). VANGL2 shows enhanced expression in 24% of invasive breast carcinomas compared to healthy tissues (39). Furthermore, increased levels of VANGL2 are linked to higher rates of recurrence and reduced metastasis-free survival in breast cancer patients (40). The protein BRCC3 is a complex component containing breast cancer type 1 susceptibility protein and breast cancer type 2 susceptibility protein. BRCC3 functions as an E3 ubiquitin ligase (41). The presence of BRCC3 is correlated with enhanced cellular proliferation and metastasis of TNBC (42). 

ANP32E, an acid nuclear phosphoprotein and a leucine-rich repeat protein family member, has diverse activities in cell adhesion, early mammalian development, and cancer metastasis (43). ANP32E has been recognized as part of a six-gene profile linked to lung metastases in the setting of breast cancer (44). Xiong et al. discovered that elevated expression of ANP32E is linked to poorer overall survival and heightened chances of disease recurrence in TNBC. This highlights ANP32E’s significance as an autonomous prognostic factor. Moreover, ANP32E stimulates the proliferation of TNBC cells by triggering the transition from the G1 to the S phase and promoting tumor formation through the transcriptional activation of E2F1 (45). Ruff et al.’s findings suggest that ANP32E may regulate breast cancer development and tumor plasticity by controlling the chromatin state (46). 

An essential role in cytokinesis has been attributed to Anilin (ANLN). ANLN resides within the nucleus during the cell cycle interphase. ANLN assembles in the cytoplasm during telophase, forming a contractile ring and cleavage furrow through interactions with various proteins, such as myosin, F-actin, RhoA, and septin (47). Research has shown that ANLN promotes the growth of cancer cells by controlling the course of the cell cycle. In instances such as ANLN-depleted breast cancer lines, the presence of polynucleated cells was detected, and cell growth was suppressed (48). Furthermore, breast cancer patients with elevated ANLN expression levels demonstrated a substantially worse overall survival rate (49). ANLN has been suggested as a prognostic marker for breast cancer; however, its relevance in TNBC and its regulatory molecular mechanisms remain unknown. Maryam et al. discovered that the expression of ANLN, driven by super-enhancers unique to TNBC, contributes to the clonogenicity of TNBC. They also identified TWIST1 and BMP2 as crucial genes that mediate ANLN’s role in maintaining stemness (50). An investigation of the functions of the VANGL2, BRCC3, ANP32E, and ANLN genes in patient outcomes, specifically as target genes of miR-548F-3p in TNBC, offers a significant understanding of the intricate regulatory networks that control these genes in TNBC. This study enhances our understanding of these genes’ clinical consequences and possible therapeutic significance in the complex TNBC landscape.

This study effectively analyzed gene expression profiles in breast cancer by leveraging robust statistical methods, such as WGCNA and PPI network analysis. However, its reliance on a single dataset (GSE76275) limits generalizability, as the findings may not represent a broader population or other subtypes. Moreover, although gene microarray data offer valuable insights, they fail to capture the complexity of gene regulation, including post-transcriptional modifications. Despite their rigor, the statistical methods could be affected by sample heterogeneity, and survival analysis is constrained by limited clinical data, potentially overlooking confounding factors. To enhance future research, incorporating data from multiple independent cohorts is recommended to improve generalizability and address confounding factors in the survival analysis. Additionally, exploring a multi-omics approach is crucial for advancing the understanding of the molecular mechanisms underlying breast cancer.

(a) Boxplot depicting the distribution of gene expression values post-normalization for the GSE76275 dataset. (b) Volcano plot illustrating the DEGs between TNBC and other breast cancer subtypes, highlighting the relationship between log fold change (logFC) on the x-axis and statistical significance (adj.P.Value) on the y-axis. (c) Evaluation of scale-free topology for weighted gene co-expression network construction, showing scale independence and mean connectivity across various soft-thresholding powers. (d) Clustering dendrogram presenting co-expressed genes, with the visual representation of gene modules identified through weighted correlation network analysis. (e) Heatmap depicting the topological overlap matrix, illustrating co-expression relationships among identified modules. (f) Venn diagram analysis revealed 118 common genes across at least two gene lists, indicating potential shared regulatory mechanisms.

(a-d). Represents BPs, CCs, MFs, and Wikipathways enrichments. The length of each bar corresponds to the *P*-value, indicating the significance level of enrichment. Longer bars represent more significant enrichments. We identified substantial enrichment of gene sets by setting a threshold of *P*-value<0.05.

Genes are depicted in rectangle format, while interactions are represented by lines connecting the nodes. Within this complex network, specific genes exhibited higher connectivity and centrality, indicating their significance in the network. The top hub genes, ranked by degree method, are highlighted in red, orange, and yellow, emphasizing their central roles in the network. These top hub genes include FOXM1 (Score: 38, Log2FC: 1.359329, Adj.P.Val: 5.31E-12), KIF23 (Score: 37, Log2FC: 1.126213, Adj.P.Val: 1.07E-10), CDC20 (Score: 37, Log2FC: 1.638208, Adj.P.Val: 2.43E-15), TYMS (Score: 35, Log2FC: 1.016491, Adj.P.Val: 2.11E-18), BUB1B (Score: 35, Log2FC: 1.120616, Adj.P.Val: 6.62E-10), BIRC5 (Score: 34, Log2FC: 1.137402, Adj.P.Val: 5.74E-11), BUB1 (Score: 33, Log2FC: 1.573514, Adj.P.Val: 2.09E-13), EZH2 (Score: 33, Log2FC: 1.233123, Adj.P.Val: 5.90E-10), ANLN (Score: 33, Log2FC: 1.182217, Adj.P.Val: 3.36E-07), and PRC1 (Score: 33, Log2FC: 1.066060, Adj.P.Val: 1.50E-08).

The miRNA-gene-pathway network illustrates the interactions between miR-548F-3p and five common genes (VANGL2, BRCC3, ANP32E, SLC16A1, and SOX4) identified across all three gene lists. The network represents the regulatory relationships between miRNA and target genes and their associations with specific pathways. The nodes in the network correspond to miR-548F-3p, the five common genes, and relevant pathways, while the edges depict the connections between them, indicating the regulatory interactions. This network provides insights into the potential roles of miR-548F-3p and its target genes in specific pathways, further contributing to our understanding of their involvement in breast cancer pathogenesis.

The analysis utilized the ULACAN online database and incorporated clinical data from breast cancer patients in the GSE76275 dataset. Both OS and RFS analyses were performed to assess the impact of gene expression on patient outcomes. Kaplan-Meier survival curves were generated using the ULACAN platform, providing a visual representation of the probability of survival over time for distinct gene expression groups. The statistical significance of survival differences between these groups was assessed using the log-rank test, with a *P*-value threshold of 0.05 considered statistically significant. Genes demonstrating a significant association with survival outcomes may be identified as potential prognostic markers.

The expression levels of the desired gene in normal and primary tumor samples were analyzed using the TCGA database within the ULCAN platform. The expression data is presented in a bar graph format, with the x-axis representing the sample groups (normal vs primary tumor) and the y-axis representing the expression level of the desired gene. The bars indicate the mean expression values and error bars may be included to represent the mean's standard deviation or standard error. (a), VANGL2, TYMS, SOX4. (b), ANP32E, BRCC3, BUB1B. (c), ANLN, B1RC5, BUB1. (d), SLC16A, PRC1, KIF23. (e), CDC20, FOXM1, EZH2

**Table 1:** Comprehensive expression details of the top 10 hub genes identified and ranked by degree method including their associated expression levels

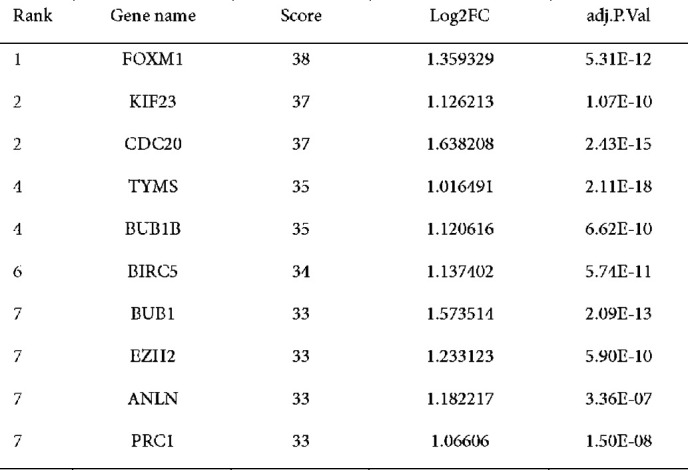

## Conclusion

In brief, our research provides a comprehensive investigation into the molecular complexities of TNBC, revealing the unparalleled significance of miR-548F-3p and shedding light on the complex regulatory networks. The spatial specificity and expression patterns unique to specific tissues highlight the potential regulatory functions that miR-548F-3p may have in different tissues. By conducting functional enrichment and pathway analyses, we highlighted the significance of spindle assembly checkpoint signaling, TNBC-specific metabolic adaptations, and mitotic spindle organization. This study establishes potentially fruitful therapeutic avenues. Notably, specific genes, including ANP32E, VANGL2, BRCC3, and ANLN, have emerged as prognostic indicators fundamental to the pathogenesis of TNBC. The comprehensive analysis of the functions of BRCC3, an E3 ubiquitin ligase associated with heightened cell proliferation, ANP32E, which regulates histones, aids in cell adhesion, and promotes the proliferation of TNBC cells, and ANLN, which facilitates cytokinesis and encourages tumor cell proliferation, highlights the critical importance of these genes in the progression of TNBC and establishes them as prospective therapeutic targets. The results above offer significant contributions to our understanding of the intricate molecular terrain of TNBC, thereby facilitating the development of targeted therapeutics and precise interventions for this particularly aggressive subtype of breast cancer.

## Data Availability

The datasets generated during and/or analyzed during the current study are available from the corresponding authors upon reasonable request.
